# A toolkit for wide-screen dynamic area of interest measurements using the Pupil Labs Core Eye Tracker

**DOI:** 10.3758/s13428-022-01991-5

**Published:** 2022-10-17

**Authors:** Yasmin Faraji, Joris W. van Rijn, Ruth M. A. van Nispen, Ger H. M. B. van Rens, Bart J. M. Melis-Dankers, Jan Koopman, Laurentius J. van Rijn

**Affiliations:** 1 Amsterdam UMC location Vrije Universiteit Amsterdam, Ophthalmology, Amsterdam, The Netherlands; 2 Amsterdam Public Health, Quality of Care, Societal Participation & Health, Mental Health, Aging and Later Life, Amsterdam, The Netherlands; 3 Royal Dutch Visio, Centre of Expertise for Blind and Partially Sighted People, Huizen, The Netherlands; 4 Department of Ophthalmology, Onze Lieve Vrouwe Gasthuis, Amsterdam, The Netherlands; 5 Amsterdam Neuroscience, Systems & Network Neurosciences, Amsterdam, The Netherlands

**Keywords:** Eye tracking, Dynamic area of interest, Visual perception

## Abstract

Eye tracking measurements taken while watching a wide field screen are challenging to perform. Commercially available remote eye trackers typically do not measure more than 35 degrees in eccentricity. Analysis software was developed using the Pupil Core Eye Tracking data to analyze viewing behavior under circumstances as natural as possible, on a 1.55-m-wide screen allowing free head movements. Additionally, dynamic area of interest (AOI) analyses were performed on data of participants viewing traffic scenes. A toolkit was created including software for simple allocation of dynamic AOIs (semi-automatically and manually), measurement of parameters such as dwell times and time to first entry, and overlaying gaze and AOIs on video. Participants (*n* =11) were asked to look at 13 dynamic AOIs in traffic scenes from appearance to disappearance in order to validate the setup and software. Different AOI margins were explored for the included objects. The median ratio between total appearance time and dwell time was about 90% for most objects when appropriate margins were chosen. This validated open-source toolkit is readily available for researchers who want to perform dynamic AOI analyses with the Pupil Core eye tracker, especially when measurements are desired on a wide screen, in various fields such as psychology, transportation, and low vision research.

**Supplementary Information:**

The online version contains supplementary material available at 10.3758/s13428-022-01991-5.

## Introduction

Eye tracking is growing in popularity amongst researchers from many different disciplines, including healthcare, psychology, biomedical applications, and neuroscience (Carter & Luke, 2020; Holmqvist & Andersson, 2017). An eye tracker measures how the gaze is directed during a specific task and can give information about the allocation of visual attention as eye movements are linked to cognitive processing. Currently, many methods are based on area of interest (AOI, also known as region of interest; ROI) analyses. AOIs are defined as areas in the stimulus important to the research aim and can be used to calculate metrics such as AOI hits (when gaze coordinates lay inside an AOI) and dwell times (duration of one visit in an AOI, from entry to exit) (Holmqvist & Andersson, 2017). Dynamic AOIs—moving areas of interest that arise during a video or animated elements on a screen—challenge the analysis since the objects move relative to the coordinate system in which the gaze position data are recorded (Hessels et al., 2018).

Some eye trackers provide software for the analysis of dynamic AOI data, such as Tobii Pro Lab. Also, open-source options are available, such as DynAOI (Papenmeier & Huff, 2010). However, a limitation of commercially available remote and tower-mounted-based eye trackers is the restriction of head movements and a limited measurement range, typically not more than 35 degrees eccentricity. Head-mounted eye trackers that allow for free head movements and have a large measurement range are available, such as Tobii Pro glasses and Pupil Core eye trackers. However, head-mounted eye trackers provide a gaze-overlaid video and if a data file is provided, the coordinates refer to positions in the video (eye-in-head coordinates). Therefore, the use of dynamic AOI analyses becomes problematic.

Our research group aims to study compensatory viewing in traffic for persons with visual field defects in the TREYESCAN study (Traffic Eye Scanning and Compensation Analyzer). The current method of visual field testing does not properly discriminate between persons with visual field defects that are fit and unfit to drive (Faraji et al., 2022). The TREYESCAN should measure eye movements over a large field of view because the visual field is important for safe participation in traffic (Owsley & McGwin Jr., 1999). Defects could lay in the periphery of the visual field, subsequently underlining the need of measuring eye movements on a screen as large as possible, instead of only centrally on a small monitor. Moreover, transportation research showed that a restricted field of view of driving scenes (presented on a single screen) may lead to poorer hazard detection and less eccentric eye movements compared to a setup with the addition of side views on adjacent screens (Alberti et al., 2014; Shahar et al., 2010). Therefore, we sought an accessible method for analyzing eye movements on a screen with a wide field of view (100°) while not restricting head movements. In order to measure compensatory viewing, we are interested in conducting dynamic AOI analyses.

The Pupil Core eye tracker (Pupil Labs, Berlin), that we used in this research, can detect apriltags (QR-like markers) (Wang & Olson, 2016), and map the gaze onto the defined surface using Pupil Labs’ Application Programming Interface (API) (Kassner et al., 2014). By placing the apriltags on the bezels of the computer screen, fixed coordinates of gaze can be calculated and be used in dynamic AOI analyses, while maintaining a wide measurement area and free head movements. In essence, the mobile eye tracker is used in such a matter, that it facilitates remote eye tracking on a much wider screen, as was previously investigated in an explorative study (Haase et al., 2019). The Pupil Core can measure up to 200 Hz per eye (120 Hz with higher resolution) and is a relatively affordable and valid option when mid-range accuracy is sufficient (Ehinger et al., 2019). Pupil Labs offers open-source software, which is relatively quick to include new developments. However, current drawbacks of this software are the absence of tools for dynamic AOI allocation and analysis software.

Therefore, our aim is to develop a toolkit for the Pupil Core eye tracker, in order to perform pre-recorded gaze analyses of dynamic AOIs on a large screen with free head movements. The kit includes tools for simple allocation of dynamic AOIs (semi-automated and manually), measurement of parameters such as dwell times and time to first entry, and overlaying gaze and AOIs on video. In this paper, we present the validation results of these tools on a group of normal-sighted participants. With our software, it will be possible to quantify viewing behavior for various purposes, especially when screen-based measurements are desired on a large screen. The source code of the entire toolkit is available on GitHub (https://github.com/treyescan/dynamic-aoi-toolkit).

## Methods

### Participants

Participants were recruited using snowball sampling at Amsterdam UMC for a validation study of the toolkit. Eligibility criteria were: age above 18 years, no history of ophthalmic comorbidities, no medication use that could affect responsiveness and concentration, and no refractive correction by means of glasses or contact lenses. Participants performed an Esterman visual field test (Esterman, 1982) and a visual acuity measurement using the Early Treatment Diabetic Retinopathy Study (ETDRS) chart (Ferris III et al., 1982; Yu et al., 2021). In addition, a custom suprathreshold visual field test performed on the Humprey Field Analyser II (HFA) to screen the central 10° for visual field defects. Only participants with a minimal binocular visual acuity of 0.0 LogMAR without refractive correction and no defects on the visual field tests were included. Participants were instructed not to wear eye makeup.

All participants provided informed consent and all procedures were approved by the Medical Ethical Committee of Amsterdam University Medical Centers—location VU University Medical Center.

### Validation task

The validation task included 13 short traffic scenes, which contained 13 dynamic AOIs varying in size, velocity, direction, and location on the screen (one object per scene). The participants were instructed to look at a certain object in each scene and track it from appearance to disappearance. Before the AOI appeared, a verbal instruction was given of the area in the scene it would appear, hence the participants already looked in that direction when the AOI appeared. The total experiment duration was 5 min. For each object, we were interested in the total dwell time and time to first entry, with the aim of validating our setup.

Videos had been recorded while driving in everyday traffic with a Sony A7III camera with a Laowa Zero-D ultra-wide field 12 mm f/2.8 lens (angle of view: 121.96°; minimal distortion). Footage was shot in 4K (3840x2160) with 25 fps. The camera was mounted centrally behind the windshield of a Toyota Prius II. A black piece of felt was put on the dashboard to prevent reflections from the dashboard surface.

**Fig. 1:**
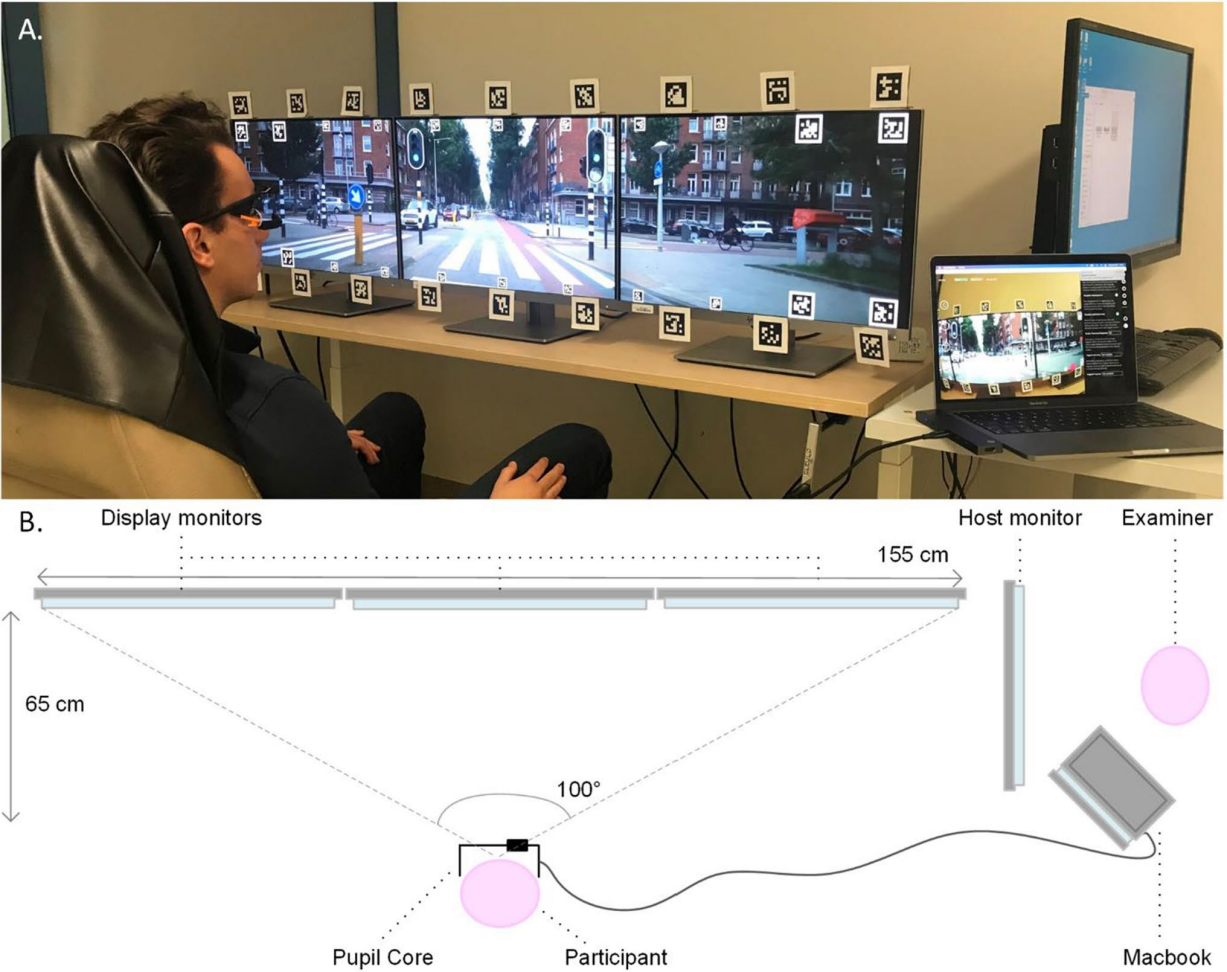
TREYESCAN setup. **a** Picture of setup. Participants are seated in front of the display monitors while wearing the Pupil Core eye tracker. **b** Schematic overview of the set-up (view from above). The participant is located 65 cm in front of the central display monitor. The examiner is located on the right of the participant and can guide the experiment from the host monitor. On the MacBook display, stability of the signal and performance of the participant can be checked

Adobe Premiere Pro (Adobe Inc, San Jose, CA, USA) was used to expand and crop the video clips to 5760x1200 to fit the three screen setup (Fig. 1). The full width of the video was used. No information about the traffic scene was lost by cropping the video’s height to facilitate screen fitting.

### Experimental setup and recording device

The validation experiment was conducted at the Amsterdam UMC, location VUmc. In a recording room, 3 HP EliteDisplay E243i 24-inch IPS LED backlit monitors with 1920x1200 resolution with thin bezels (width bezel: 0.68 cm) and a refresh rate of 60 Hz were placed in a linear formation. Nvidia Surround (Nvidia, Santa Clara, CA, USA) was used to span the video over the three screens with bezel correction. To view the video without distortion, a correction of 50 pixels was applied between two adjacent screens, thus occluding two minor portions of the video. An additional screen (Iiyama ProLite XB2783HSU) was placed on the side, the display not visible to the participant, in order to control the validation experiment. A car seat was positioned in front of the three screens at a distance of 65 cm from eyes to the central screen’s middle, in order to obtain a 100° field of view, which confines the possibility for a larger distance to the screen. The table could be altered in height to ensure the eyes were positioned in the middle of the screen (Fig. 1). Head movements were permitted in all directions.

The participants’ eye movements were recorded by a head-mounted eye-tracker (Pupil Labs Core glasses, received October 2021, Pupil Labs, Berlin, Germany). The Pupil Labs eye-tracker (Kassner et al., 2014), used in this study, has three cameras: one world camera (100° fisheye field of view, 30-Hz sampling frequency, resolution on a subset of 1280x720 pixels) to record the world from the participant’s point of view, and one eye-camera for each eye (120-Hz sampling frequency, resolution on a subset of 400x400 pixels). Pupil Labs capture v3.3.0 was used for the recordings. The experiment was conducted using two computers: Acer Nitro (N50-610 I9426-JK with NVIDIA GeForce RTX 2060 video card and Intel Core i5 processor) for stimulus presentation and an Apple MacBook Pro with M1 chip (as recommended by Pupil Labs) for recording of eye movements. All experiments were performed under the same lighting conditions (~ 250 Lux). The luminance differs for the central screen (1–210 cd/m^2^) and the peripheral screens (1.5–150 cd/m^2^), measured from the position of a participant.

Before each validation task, a nine-point screen calibration routine was used on the three-screen setup as provided by Pupil Labs (personal correspondence). Similarly, the calibration was validated by a routine with 12 points of different positions. The Pupil Labs software then generates a value for the accuracy and precision. The validation routine was repeated after the task, to get insight in changes in accuracy and precision throughout the task, such as slippage of the glasses (Niehorster et al., 2020).

### Methods of data analysis

Pupil Labs Player v3.3.0 was used to export the measurements. The analysis script was written in Python 3.8.3 (Van Rossum, 1995) using NumPy (Harris et al., 2020), pandas (McKinney, 2010), OpenCV (Bradski, 2000), and SciPy (Virtanen et al., 2020). For visualization, Matplotlib (Hunter, 2007) was used.

#### Surface definition

**Fig. 2:**
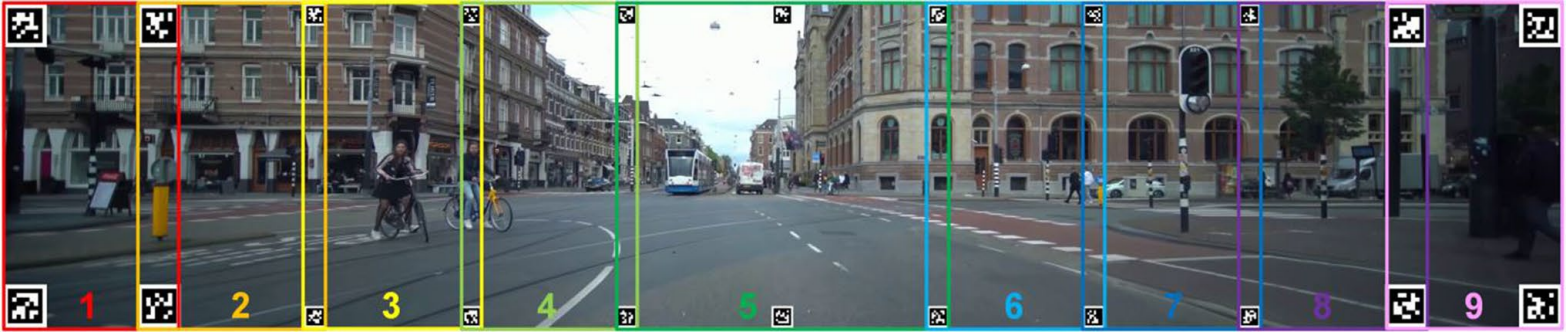
Indication of the surface distribution

**Fig. 3:**
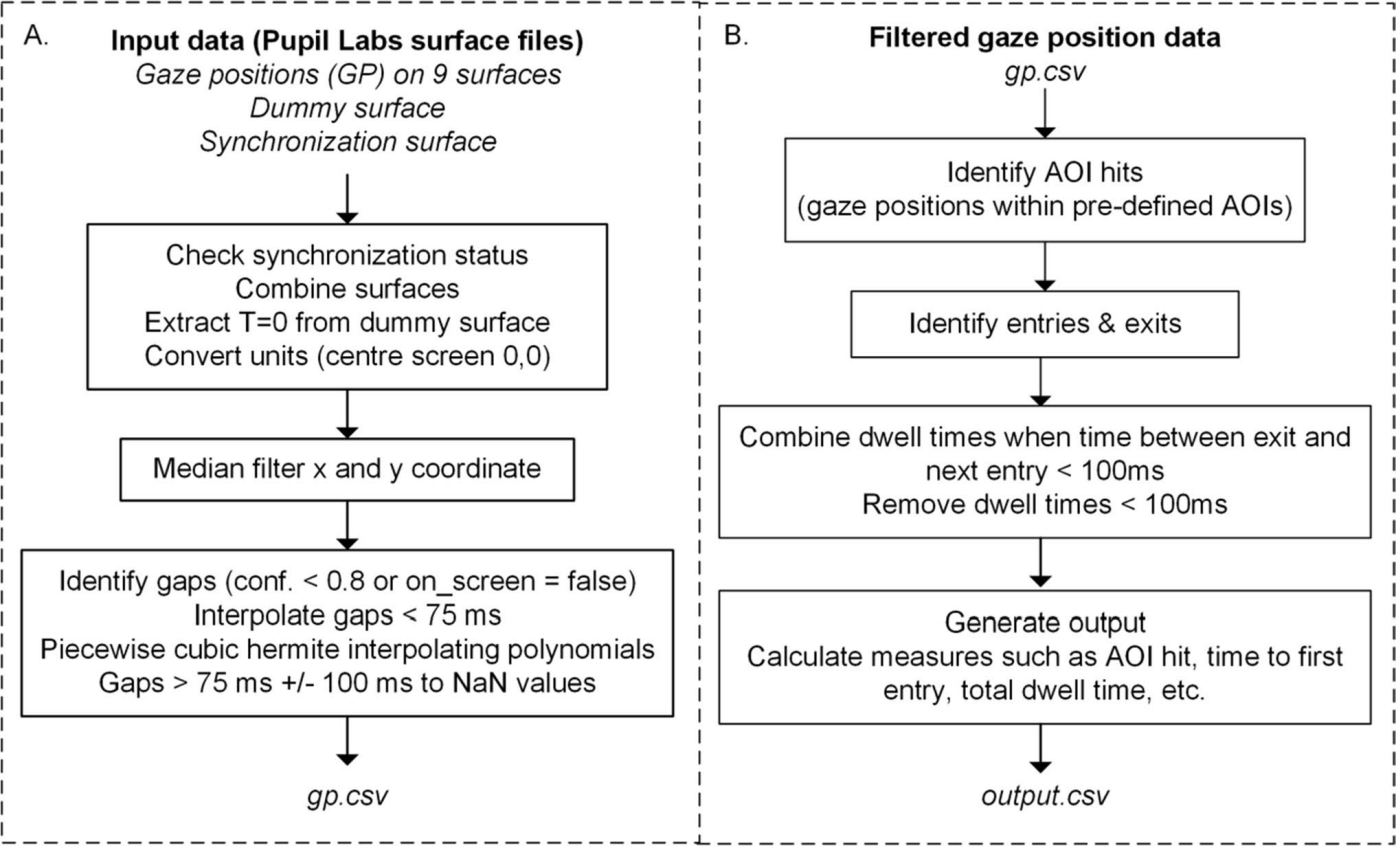
Flowcharts of steps in data processing. **a** Pre-processing of the gaze data. **b** Gaze and AOI matching process

The Surface Tracker plugin by Pupil Labs (Kassner et al., 2014) was used to define the surface area of the display with apriltags (Wang & Olson, 2016). Because of the wide screen, the surface was divided in nine Pupil Labs surfaces (Fig. 2). We found that with more and narrower surfaces the gaze coordinates became more accurate. Twenty-two apriltags were placed within the video (width of 80 pixels). The corner apriltags were enlarged for better detection at greater angles (width of 160 pixels), as seen in Fig. 2. A Python routine is included for this purpose in the toolkit. The surface detection was not constantly optimal due to the small size of the apriltags and the backlight of the screens, hence 18 additional apriltags printed on paper (width marker of 4 cm, total width including white border of 6 cm) were placed on the top and bottom row of the screen’s bezels in order to enhance the detection quality of the surfaces (Fig. 1). A dummy surface was defined, merely registering the screen’s apriltags, in order to benchmark the start and end of the task. In between each scene, an additional unique apriltag was placed in order to monitor the time synchronization of the task and obtain an indication of possible latencies. The nine surface gaze files were pooled to one gaze file (Fig. 3a) and the coordinate system was transformed so that the screen’s middle was (0,0).

#### Pre-processing the gaze data

A median noise reduction function was used as a low-pass filter on the eye movement data to smooth out noise (Fig. 3a), while preserving the features of the sampled data (Juhola, 1991). We chose a median noise reduction algorithm, because compared to a moving average algorithm, the data is less smoothed even though the most prominent noise is removed, less ‘false’ gaze coordinates are created and the amplitude of the velocity peaks is not reduced as severely (Olsen, 2012). A window size of three samples was chosen for one-sample spike reduction (Larsson et al., 2016).

Pupil Labs provides a quality assessment of the pupil detection for every sample, as a “confidence” value between 0.0 (pupil could not be detected), and 1.0 (pupil was detected with very high certainty). A Boolean variable (on_screen) is also provided by Pupil Player which indicates if the gaze was plotted within the surface areas. In our software, samples with a confidence level below 0.8 (e.g., because of blinks) and samples outside the monitor’s surface were treated as gaps in the data (Fig. 3a). This threshold is also used by Pupil Labs when determining valid gap samples for the calibration procedure. If the gap duration was below 75 ms, the gaze coordinates were filled in using linear interpolation (Komogortsev et al., 2010; Olsen, 2012). Longer gaps were kept in the data frame and labeled as Not a Number (NaN) values. We regarded the samples ± 100 ms around a gap as additional gap samples, where the pupil of the eye may be partially occluded (Costela et al., 2014).

The Pupil Core eye tracker has two eye camera’s that each measure with a sampling rate of 120 Hz in anti-phase. The Pupil Labs Fusion algorithm combines these signals to a sampling rate of 240 Hz. However, as it does not provide a constant sampling rate, we used piecewise cubic Hermite interpolating polynomials to obtain samples at a sampling rate of 240 Hz (Ehinger et al., 2019).

#### AOI allocation

In order to determine if a participant viewed an AOI, the coordinates of the bounds of these AOIs must be obtained. We decided to determine all AOIs by the use of rectangles, as this was the most accessible shape for the AOIs in traffic scenes.

Two programs, written in Python, were used to draw rectangles around the 13 dynamic AOIs in the traffic scenes. AOI_tracking.py tracks the object semi-automatically by using an OpenCV algorithm, which compares to consecutive frames and redraws the AOI on the next frame. It also corrects the size of the rectangle according to the size of the AOI, as objects that move away or towards the camera vary in size considerably from beginning until the end. We noticed this program works well, except in some instances, e.g., when an object covers a large part of the scene. Hence, we also created another script that interpolates the bounding boxes between two boxes drawn, AOI_selection.py. This program also contains an option to draw AOIs entirely manually frame by frame. Both scripts generate a bounding box with *x* and *y* values for each object on the relevant frame number, an object type label and allow for additional custom labels. The coordinates of the AOI bounding boxes are also transformed to the coordinate system with (0,0) as the screen’s center.

#### Matching gaze and AOI data

A frame-by-frame method was used to match the gaze data with the AOI data (Fig. 3b). We computed the corresponding frame number for each eye tracker sample, since the eye tracker samples were retrieved with 240 Hz (120 Hz for each eye camera) and the AOI data was based on footage with 25 fps. For each gaze sample, we checked if the corresponding frame number lay within the boundaries of the AOI box.

A variable margin (in degrees of visual angle) can be added around every AOI box in order to compensate for eye-tracking inaccuracy (Holmqvist & Andersson, 2017; Orquin et al., 2016). Because of the large angle of view (100°) these margins, in screen coordinates, become larger at the peripheral parts of the screen. This is calculated frame-by-frame, by determining the distance in pixels for the left and right side of the AOI separately for a given degree, as these can be significantly different due to the location and size of the AOI on the screen. For the top and bottom, both margins are calculated using the center *y*-coordinate of the AOI. It is recommended to add a margin around AOIs of 1° to 1.5° of visual angle, and when accuracy is low, increase margin size to ensure inclusion of all fixations on an AOI. However, larger AOI margins increase the risk of attributing fixations that do not belong to an object (Holmqvist & Andersson, 2017; Orquin et al., 2016). Therefore, for the results of the validation task we experimented with different values for the margins to give insight in the effects on parameters such as dwell time percentages.

The entries and exits within an AOI were extracted and the dwell times between each entry and exit were calculated. We assumed the time between an exit and a new entry should not be shorter than 100 ms, because this would likely be due to precision errors than of the participant’s gaze deliberately exiting and entering the object. If this time was indeed shorter than 100 ms, the time between the exit and new entry was pooled with the previous dwell time, thus combining the two visits. If a dwell time remained shorter than 100 ms, the dwell was not included in the total dwell time measure. The sum of dwell times provides the total dwell time within an AOI. We decided on 100 ms as a threshold for these variables, since Engmann et al. (2009) found that 96.1% of the fixations in their study lasted longer than 100 ms. Also, Salvucci and Goldberg (2000) report that fixations typically have a duration of at least 100 ms. For a dwell time to be relevant it needs to consist of at least one fixation otherwise no cognitive processes could have taken place. Thus, 100 ms was considered a safe cut-off. However, the values of these variables can be altered in the toolkit according to the experiment’s requirements.

Time to first entry is computed by extracting the time between the objects first appearance and the first entry. The dwell time percentage is calculated between the total appearance time of an object and the total dwell time to explore what percentage of time the object was looked at when it was in view.

#### Overlay gaze and AOIs in video footage

We developed three tools for overlaying the gaze over the video for visualization of included AOIs and gaze data. The tool *overlay_aois.py* overlays the drawn AOI bounding boxes with margins, *overlay_single_participant.py* overlays all AOI boxes with the gaze data of one participant and *overlay_multiple_participants.py* overlays all AOI boxes with the gaze data of all participants. For the overlay tools discriminative colors were used from the color alphabet (Green-Armytage, 2010).

#### Statistical analysis

The statistical analyses were performed with IBM SPSS Statistics for Windows, Version 28.0 (SPSS, Armonk, NY, USA) and our analysis software programmed in Python. Graphs were made in GraphPad for Windows, Version 9.0 (GraphPad Software, San Diego, CA, USA).

## Results

Eleven participants (median age 27, range 25–59, 5 female) were included to perform the validation task. The participant and measurement characteristics are shown in Table 1. Accuracy and precision, as provided by Pupil Labs and obtained during the validation procedures before and after the task, did not change significantly during the task of 5 min.

**Table 1:** Participant and measurement characteristics

	*n* = 11
Age (year) median [range]	27 [25–59]
Number of female participants (*N*)	5
Binocular visual acuity (logMAR) mean ± SD	0.13 ± 0.091
Esterman visual field abnormalities	0
Prescription eyeglasses/contact lenses	0
Accuracy before task (°) mean ± SD	2.05 ± 0.43
Precision before task (°) mean ± SD	0.10 ± 0.017
Accuracy after task (°) mean ± SD	2.12 ± 0.69
Precision after task (°) mean ± SD	0.098 ± 0.020

When pre-processing the gaze data, samples with a confidence value below 0.8 were set to NaN values. Before interpolation, this was 3.59% (median, IQR [1.87–11.08]) of total samples. 2.14% (median, IQR [1.44–3.88]) of total samples were marked as samples outside the monitor’s surface and were also set to NAN. Some samples had both a poor confidence and were not on screen, hence after this step, a total of 5.20% (median, IQR [2.75–13.56]) of samples were set to NaN values. Subsequently, the length of the gaps was determined and gaps that were shorter than 75 ms were interpolated. After this interpolation, 3.89% (median, IQR [2.56–7.98]) of samples remained a NaN value. When extending the gaps with ± 100 ms, 7.05% (median, IQR [5.01–14.16]) of samples were set to NaN values. These samples were consequently considered as gap samples in the analyses.

As can be seen in Table 2, objects of various sizes, location, direction, and velocity were included in the validation task. An object can have a large range of sizes, as the objects become larger when nearing the camera, e.g., Road Sign 1, which starts as a small object and becomes a large object at the side of the screen when the car passes it. We included objects that move from one side of the screen to the other, and objects that appear in the center of the screen and disappear from the sides, which is the case for oncoming traffic, traffic lights, road signs etc. Only Car 2 starts in the middle and ends in the middle. Van 1 stands out as an object with a short and rapid appearance.

**Table 2:** Traffic scenes and AOI characteristics

	Duration of scene (s)	Duration of AOI visibility (s)	Width AOI (pixels; median (min – max))	Height AOI (pixels; median (min – max))	Average velocity of AOI (°/s)
Car 1	17.6	10.5	865 (150–2350)	275 (75–725)	7.2
Car 2	69.4	69.2	168 (48–544)	144 (52–284)	0.02
Cyclist 1	23.2	9.8	160 (70–525)	200 (125–455)	9.7
Cyclist 2	17.3	9.8	215 (85–660)	390 (140–660)	8.6
Cyclist 3	11.6	4.6	160 (60–590)	165 (90–540)	11.6
Cyclist 4	13.2	10.3	52 (40–1020)	120 (76–1168)	4.6
Pedestrian 1	31.6	25.5	195 (70–455)	210 (75–330)	2.0
Road Sign 1	10.8	6.4	60 (40–892)	68 (36–424)	6.9
Road Sign 2	11.2	6.8	44 (24–185)	42 (28–245)	7.1
Scooter 1	23.2	9.8	104 (24–185)	160 (24–185)	9.0
Scooter 2	9.3	5.4	36 (28–1116)	72 (44–920)	8.1
Traffic light 1	12.6	11.8	32 (20–340)	60 (40–410)	3.7
Van 1	11.6	1.7	1790 (160–2110)	730 (395–760)	56.5

Width of screen: 5760 pixels. Height of screen: 1200 pixels. Duration of Scene: the duration of the entire scene in which the AOI is included. Duration of AOI visibility: the time during which the AOI is visible. The average velocity is calculated by dividing the trajectory (middle of start point till middle of end point) in degrees by duration of AOI visibility (negative numbers represent objects that move from the right to the left side of the screen)

**Fig. 4:**
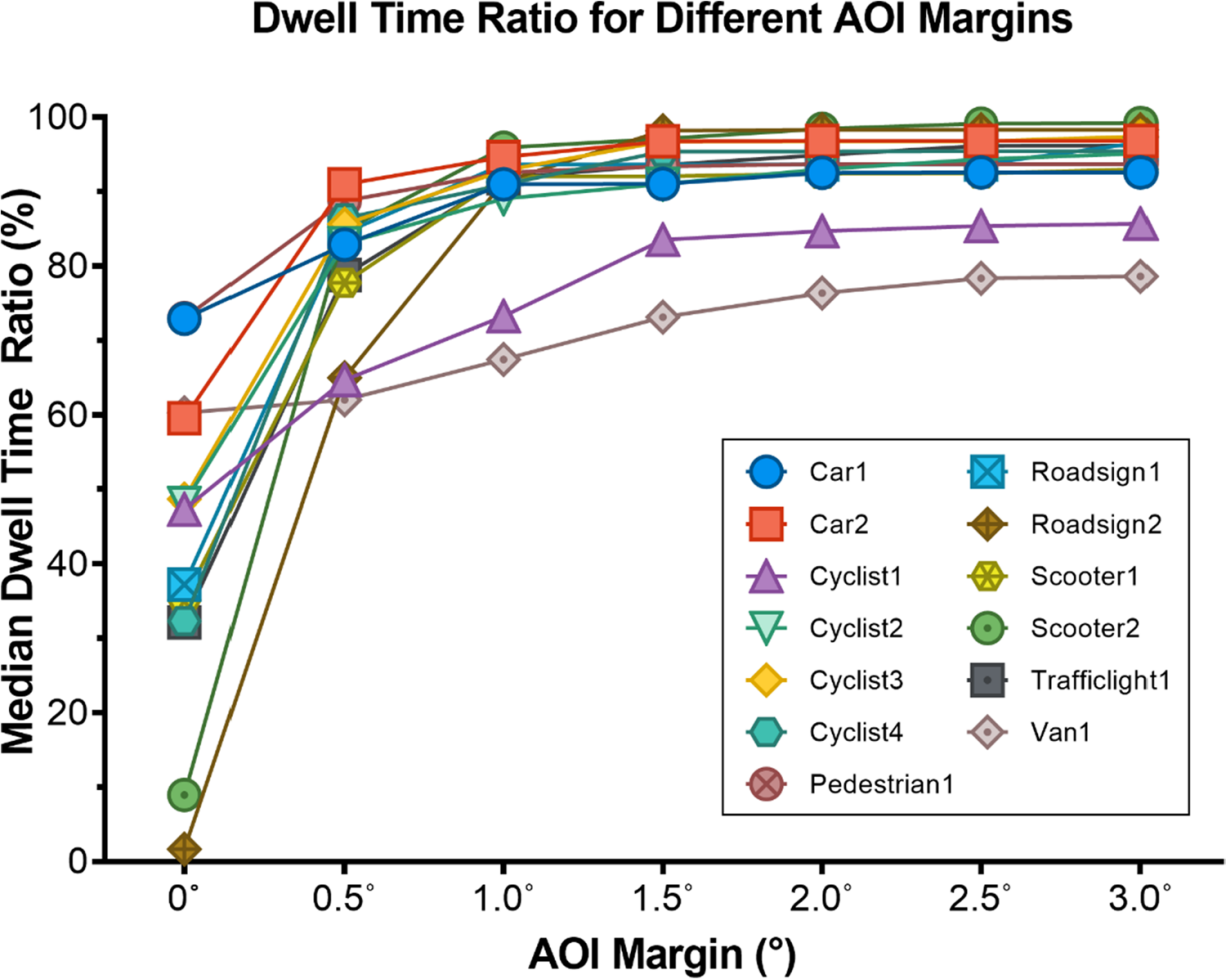
Median dwell time percentage for different AOI margins. Each *symbol* denotes the median dwell time percentage for 1 AOI

Our toolkit was applied on the gaze data for different sizes of AOI margins. Figure 4 presents the percentage between total appearance time and calculated dwell time for each object. It shows that adding a margin, considerably increases the calculated dwell time percentage. After 1.5° the measures only improve slightly and most dwell percentages lay around 90%. This margin seems the most acceptable margin, as it is large enough to reduce inaccurate hits, but is at the same time the smallest margin possible to decrease possible overlap with other AOIs in the scene. In the supplementary material S1 (Video Van1) and S2 (Video Scooter1) two objects of the task can be seen with the gaze data and AOIs with margins of 1.5°. Also, the raw data table that supports Fig. 4 is included in S3.

**Fig. 5:**
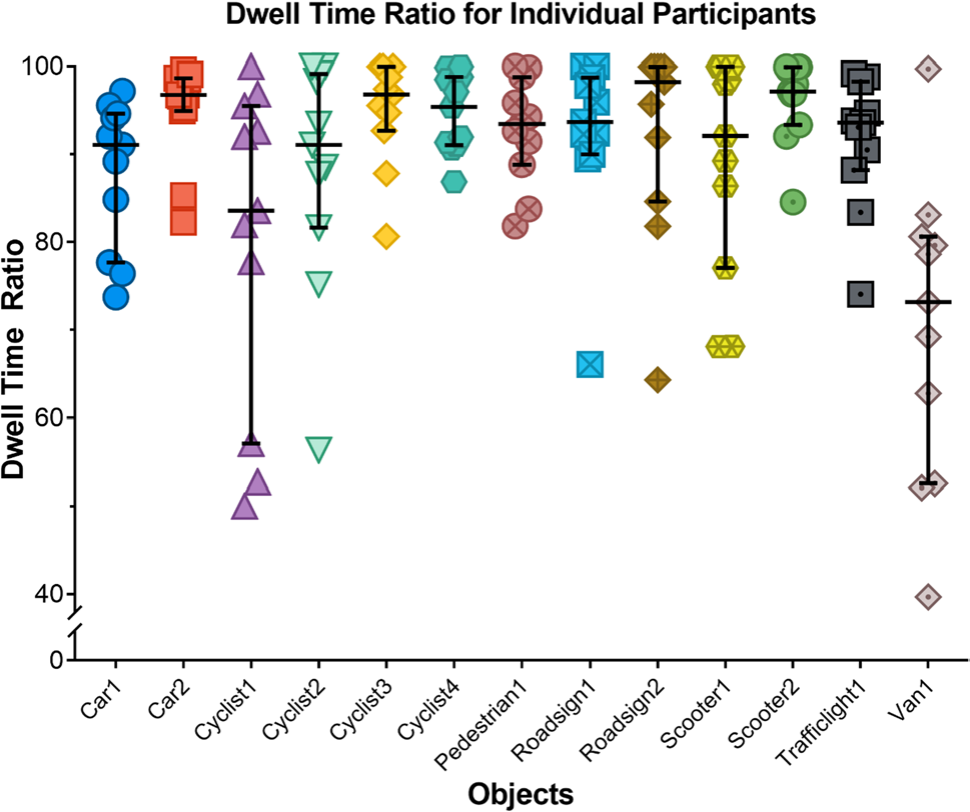
Dot plot of the dwell time percentage (%) for each object for AOI margin 1.5°. Each *dot* represents one participant. The median and interquartile range are also illustrated

Figure 5 gives insight in the distribution of calculated dwell time percentages for each participant. It can be seen that when a margin of 1.5° is chosen, most calculated dwell time percentages of the participants are above 80% and median dwell time percentages are around 90%. Especially Van 1 and Cyclist 1 show a wide distribution of dwell time percentages.

The median time to first entry measure was close to zero and below 50 ms for most objects, when a margin of 1.5° is chosen. However, the last exit time was often significantly earlier than the disappearance time for the objects that disappear at the sides of the screen, which is the case for most objects.

### Feasibility

The question is why we did not obtain dwell time percentages of (close to) 100% for all participants when viewing the AOIs, since participants were instructed to track them from appearance to disappearance. Multiple factors could contribute to our results.

Gap samples (during blinks) were excluded from the dataset. When an AOI is viewed for an extended period of time such as Car 2, it is inevitable that the participant will blink multiple times, which has an effect on the measured dwell time.

Furthermore, when analyzing the results of our toolkit we assumed that participants had followed the objects accordingly and that discrepancies were due to eye tracker or toolkit inaccuracies. However, we noticed that especially fast moving objects, such as Van1 (see S1 – Video Van1) are not properly followed by all participants. In some instances, a participant did not immediately look at an object after it appeared (although instructed otherwise).

When looking at the gaze data of the validation task it is clear that the accuracy gets poorer towards the sides of the screen at larger angles, between 40 and 50° (see S2 – Video Scooter1). It is well known in eye-tracking research that accuracy is best in the middle and poorest in the corners of the screen (Holmqvist & Andersson, 2017). We aimed to correct for this by adding a larger margin towards the sides of the screen as determined by the position of the object on the screen, but this was not enough for some cases.

Furthermore, when the participant looks at the side of the screen, due to the inaccuracy at larger angles, data samples are labeled as “not looking on the screen” and hence considered as missing samples in the dataset. This is visible in the Video Van1 (see S1) where the gaze points of multiple participants are not visible because the gaze points fall outside of the screens edge due to the inaccuracy. We assume that this aspect was the biggest issue for the lower dwell time percentage, since most objects disappear from the sides of the screen. We expect this not to be a disturbing issue when using this method on a natural viewing task, because then participants will not be instructed to follow an object from appearance to disappearance, and generally will not be looking at the edges of the screen.

## Discussion

In this paper, we present a toolkit for analyzing dynamic AOI analyses on a large screen without the restriction of head movements. The results of our validation task show promising results for the functionality of the toolkit using the Pupil Core eye tracker. For most followed objects (11/13) the calculated median dwell time percentages are around 90%.

When using video stimuli in eye tracker research, synchronization between stimulus presentation and recording software is critical (Holmqvist & Andersson, 2017). As we presented the stimuli and the recording software on different computers, the probability of latencies decreases, because the processor and hard disk do not have to perform the demanding operations simultaneously. However, the probability of latency remains, because video players typically run slightly faster or slower than the recording of data samples, and as a result the data sample resulting from a participant looking at a particular frame in the video can be stored earlier or later in the data file. Moreover, the beginning of the recordings is determined by the presentation of the apriltags. Since the scene camera records in 30 fps, a delay of 33 ms could occur after the onset of the video. In order to get an indication of the synchronization status in our setup, we placed an apriltag between every scene of the validation task. We found a discrepancy of 0 to 62.5 ms between the expected appearance of the apriltag and the actual eye-tracking data. When also considering the inherent delay of 10 ms of the Pupil Labs’ cameras (Ehinger et al., 2019), we find this value acceptable for our research aim.

In some eye-tracking studies, researchers use fixation measures instead of dwell times measures for AOIs analyses. In this toolkit, we decided to use dwell time measures for the dynamic areas of interest analysis. Susac et al. (2019) concluded that it is adequate to report only one of these measures. In addition, Vansteenkiste et al. (2015) found a high correlation when comparing a fixation-by-fixation analysis to a frame-by-frame method when analyzing dwell time percentages. This indicates that both methods work well. A frame-by-frame method was chosen for our toolkit to check for robustness without adding another subjective variable. However, the fixation algorithm provided by Pupil Labs can also be used with our detection software.

In this toolkit we offer two tools for the allocation of AOIs. Recently, Bonikowski et al. (2021) also presented open-source software for determining dynamic AOI using object tracking. They offer a neat application with integrated control panels. A benefit of our toolkit is the possibility of margin addition and the incorporation of the software that matches gaze with AOIs.

A limitation of our validation study is the inclusion of individuals without glasses or contact lenses. We wanted to test our toolkit in the most ideal situation. Hence, no conclusions can be drawn about the functionality of the Pupil Core eye tracker in combination with our toolkit when using refractive correction. The AOIs were in all cases defined as rectangles, while AOIs such as round traffic signs and cyclists, do not fill the rectangle shape entirely. However, this was the most accessible shape for most objects in traffic situations. Moreover, the results are based on a small sample of 11 participants, who watched scenes for 5 min, which may not represent the accuracy and precision during a longer task. The analysis of dynamic AOIs remains complex. Since objects move relative to the coordinate system in which the gaze position data is recorded, it is difficult to make any definite statements relating to the size of the AOIs.

When considering all the challenges that arise with this type of measurement methods and analyses, it can be concluded that our toolkit performs acceptably for the research aim. To the best of our knowledge, this is the first toolkit that uses the Pupil Core eye tracker and apriltags for dwell time measures in dynamic areas of interest on a large screen. As well as providing various tools that are necessary for analysis and visualization purposes.

## Conclusions

This validated open-source toolkit is ready to use for researchers who want to perform dynamic AOI analyses with the Pupil Core eye tracker, especially when measurements are desired on a wide screen. We provide tools for simple allocation of dynamic AOIs (semi-automatically and manually), measurement of parameters such as dwell times and time to first entry, and overlaying gaze and AOIs on video. With our software, it is possible to quantify viewing behavior for various purposes. In further research, our aim is to investigate compensatory viewing strategies in traffic, but our setup is also readily available for eye tracking studies in other fields, such as psychology, transportation, and low vision research.

### Supplementary information

ESM 1 (MP4 3775 kb)

ESM 2 (MP4 5208 kb)

ESM 3 (DOCX 16 kb)

## Data Availability

The raw eye tracking data is made available in an online depository (10.17026/dans-zke-zccc), this data can be used with the source code to test the toolkit.
