# Supplementary material for: A toolkit for wide-screen dynamic area of interest measurements using the Pupil Labs Core Eye Tracker
Source: Behav Res Methods. 2022 Oct 17;55(7):3820–30. doi: 10.3758/s13428-022-01991-5 (PMC10616213; doi:10.3758/s13428-022-01991-5)
Supplement: Supplementary file 3 — (DOCX 16 kb) [file 13428_2022_1991_MOESM3_ESM.docx]

S3 Table Calculated Dwell Time Percentages

Table S3. Calculated dwell time percentage (%) for different AOI margins (median (IQR))

|  | *0°* | *0.5°* | *1.0°* | *1.5°* | *2.0°* | *2.5°* | *3.0°* |
| --- | --- | --- | --- | --- | --- | --- | --- |
| Car1 | 72.9 (63.0-88.9) | 82.9 (73.9-91.3) | 91.0 (77.7-94.0) | 91.1 (77.7-94.6) | 92.6 (77.9-95.6) | 92.6 (78.8-95.6) | 92.6 (79.8-95.6) |
| Car2 | 59.5 (39.5-75.8) | 91.0 (70.0-97.4) | 94.7 (89.7-98.5) | 96.8 (94.9-98.7) | 96.8 (95.0-98.7) | 96.9 (95.1-98.7) | 96.9 (95.1-98.7) |
| Cyclist1 | 47.3 (25.4-57.6) | 64.7 (38.2-87.8) | 73.3 (49.0-93.5) | 83.6 (57.1-95.5) | 84.7 (61.0-96.0) | 85.4 (66.5-96.0) | 85.7 (79.4-96.0) |
| Cyclist2 | 48.1 (35.2-75.3) | 83.1 (61.3-91.7) | 89.1 (76.1-97.2) | 91.1 (81.6-99.1) | 93.0 (82.5-100.0) | 94.4 (82.5-100.0) | 95.2 (82.5-100.0) |
| Cyclist3 | 48.7 (31.5-76.4) | 85.7 (73.6-92.7) | 92.9 (87.8-96.8) | 96.8 (92.7-100.0) | 96.8 (92.7-100.0) | 96.8 (94.4-100.0) | 97.4 (94.7-100.0) |
| Cyclist4 | 32.3 (18.8-58.5) | 86.5 (47.1-92.7) | 91.0 (82.9-98.8) | 95.4 (91.0-98.8) | 95.4 (91.5-99.2) | 95.5 (92.0-99.4) | 95.5 (92.0-99.4) |
| Pedestrian1 | 73.0 (44.2-82.6) | 88.8 (77.7-97.4) | 92.6 (83.8-98.3) | 93.4 (88.8-98.8) | 93.7 (88.8-99.8) | 93.7 (88.8-99.8) | 93.7 (88.8-99.8) |
| Roadsign1 | 37.3 (19.2-54.9) | 84.5 (73.1-87.2) | 93.7 (87.3-98.7) | 93.7 (90.0-98.7) | 93.7 (91.5-98.7) | 93.7 (91.5-98.7) | 96.6 (92.8-99.2) |
| Roadsign2 | 1.7 (0.1-7.1) | 65.0 (26.8-85.1) | 91.4 (80.3-97.1) | 98.2 (84.6-99.9) | 98.4 (84.7-99.9) | 98.4 (87.6-99.9) | 98.4 (89.6-99.9) |
| Scooter1 | 35.1 (23.5-42.9) | 77.8 (44.8-97.1) | 92.1 (71.7-99.1) | 92.1 (77.0-100.0) | 92.4 (85.9-100.0) | 92.5 (87.7-100.0) | 93.0 (87.8-100.0) |
| Scooter2 | 9.0 (2.9-20.5) | 84.8 (70.3-93.8) | 95.9 (89.0-99.2) | 97.2 (93.4-99.9) | 98.5 (96.0-99.9) | 99.2 (97.6-99.9) | 99.2 (97.6-99.9) |
| Trafficlight1 | 32.2 (9.0-41.6) | 78.8 (68.2-90.5) | 91.8 (82.6-96.2) | 93.6 (88.2-98.3) | 94.9 (88.2-98.3) | 96.2 (88.2-98.3) | 96.2 (88.2-98.6) |
| Van1 | 60.3 (51.8-80.6) | 62.0 (52.1-80.6) | 67.5 (52.1-80.6) | 73.2 (52.6-80.6) | 76.4 (53.6-80.6) | 78.4 (54.1-81.6) | 78.6 (54.1-83.1) |
